# A Practical Treatise on the Diseases of Children

**Published:** 1846-11

**Authors:** 


### BIBLIOGRAPHICAL NOTICES.

---

Art. II.—*A Practical Treatise on the Diseases of Children.* By James Milman Coley, M.D., Member of the Royal College of Physicians in London, &c.; author of "A Treatise on the Remittent Fever of Infants," &c., and of "Essays on Phlegmonous Erysipelas," on "Schirroide, Keloide, or Cancroid, &c., &c. Philadelphia: Ed. Barrington & Geo. D. Haswell. 1846. 8 mo.

From a cursory examination, which is all that we have had leisure to give this work, we are ready to believe that it is the most thorough treatise hitherto published in this country on the diseases to which it relates. In his introduction the author says: "The principal object I have had in view in writing the following pages, has been to present to the Medical Profession and the Public a comprehensive work on the Diseases of Infants and Children, which the physician, the surgeon, and the general practitioner may consult as a work of reference. The practical portions have been the result of an experience of forty years, during which time I have been constantly engaged in recording cases, accumulating facts, and pursuing pathological inquiries. In addition to the various information afforded by my private practice, the numerous and interesting diseases occurring among the poor of an extensive district, to whom I was in the habit of giving gratuitous advice and assistance, either at my own residence, or at an infirmary and dispensary, which I established in my native town, supplied me with opportunities rarely enjoyed for prosecuting the useful profession, to which my whole life has been devoted."

We shall take an early opportunity to express a more matured opinion of this work; meantime, as one of the volumes of the "*Select Medical Library*," it will find its way into the hands of many of our readers.

